# Surgical tactics in fire kidney injury and the first experience in performing laparoscopic nephrectomy at the II level of medical support (role II) in combat conditions: Case report

**DOI:** 10.1016/j.ijscr.2023.108046

**Published:** 2023-04-15

**Authors:** Kostyantyn Gumeniuk, Igor Lurin, Oleksandr Savytskyi, Volodymyr Nehoduiko, Vitaly Makarov, Kostiantyn Smolianyk

**Affiliations:** a Command of the Medical Forces of the Armed Forces of Ukraine, 6 Povitroflotsky Ave., 02000 Kyiv, Ukraine; b National Academy Medical Sciences of Ukraine, 12 Hertsena st., 04050 Kyiv, Ukraine; c Ukranian Military Medical Academy, 45/1, Kniaziv Ostrozkykh St., Kyiv 01015, Ukraine; d Military Medical Clinical Center of the Northern Region, 5 Kultury st., 61058 Kharkiv, Ukraine; e Kharkiv National Medical University, 4 Nauki av., 61022 Kharkiv, Ukraine; f State Scientific Institution “Scientific and Practical Center of Preventive and Clinical Medicine” of the State Administration of Affairs, 5 Upper st., 01014 Kyiv, Ukraine

**Keywords:** Kidney, Gunshot wound, Laparoscopic nephrectomy, Combat conditions, Case report

## Abstract

**Introduction and importance:**

According to the data from the American Urological Association (AUA) and the European Urological Association (EAU) (2020), kidney is the most frequently damaged organ of the genitourinary system. Kidney damage occurs in approximately 5 % of injured people and accounts for 24 % of traumatic injuries to abdominal organs. Surgical treatment remains the gold standard in unstable patients with gunshot and stab wounds. Minimally invasive surgical treatment of kidney injuries, which is usually performed after laparoscopic diagnosis, at the II level of medical care becomes possible in the first hours after injury.

**Case presentation:**

We performed two laparoscopic nephrectomies caused by gunshot shrapnel damage to the kidney in a military mobile hospital at the II level of medical support. The time since the injury was 64 ± 16 min. The wounded were extubated after the operations, activated on the first day. In one case, the drain was removed on the third day, in the other – on the fourth day. During the monitored period (30 days) after the operation, there were no complications in both wounded.

**Clinical discussion:**

Laparoscopic nephrectomy in gunshot damage to the kidney was characterized by presence of a retroperitoneal tense hematoma. When opened, there were signs of bleeding from the kidney parenchyma, difficulty of anatomical visualization of anatomical structures - ureter, renal artery and vein.

**Conclusion:**

It is possible to perform endovideosurgical operations - laparoscopic nephrectomy in combat kidney injury at a military mobile hospital with available high-tech equipment at the II level of medical support (Role II), thus bringing highly specialized care closer to the wounded. We noted a better cosmetic effect after the laparoscopic operations.

## Highlights

• FAST sonography has low sensitivity and specificity for kidney injury.
• In penetrating renal injury, contrast-enhanced CT was indicated in all patients.
• Diagnostic laparoscopy was of the greatest value for kidney injury.
• We performed laparoscopic nephrectomies due to gunshot damage to the kidney.

## 1 Introduction

According to data from the American Urological Association (AUA) and the European Urological Association (EAU) (2020), kidney is the most frequently damaged organ of the genitourinary system. Kidney damage occurs in approximately 5 % of injured people and accounts for 24 % of traumatic injuries to abdominal organs [1], [2], [3], [4]. Of the 16,323 hospitalizations of military personnel with injuries listed in the Joint Theater Trauma Registry (USA) between October 2001 and January 2008, 819 (5 %) had one or more genitourinary injuries, 65 % due to explosions. The average age of the wounded was 26 years (from 18 to 58), 98.5 % were men. 887 injuries were distributed as follows: portal vein - 257 (29.0 %); kidneys - 203 (22.9 %); bladder - 189 (21.3 %); penis - 126 (14.2 %); testes - 81 (9.1 %); ureter - 24 (2.7 %); and urethra - 7 (0.8 %). Of 203 patients with kidney damage, 22 % were admitted to the operating room, 31 patients underwent nephrectomy [5].

Surgical treatment remains the gold standard in unstable patients with gunshot and stab wounds [6], [7], [8], [9], [10], [11], [12], [13], [14], [15], [16]. Urologists prefer to save the organ, while emergency surgeons consider stabilization of the patient's condition during the “golden hour” more important than organ preservation [17], [18], [19]. It is the integrated approach and active cooperation between the two specialties that forms the basis for achieving optimal treatment and better results [19]. Minimally invasive surgical treatment of kidney injuries, which is usually performed after laparoscopic diagnosis, at the II level of medical care becomes possible in the first hours after injury.

This case report was prepared according to the SCARE Criteria [20].

## 2 Materials and methods

According to the American Association for Surgery of Trauma (AAST) and the European Association of Urologists (EAU) (2020), there are 5 degrees of kidney damage severity (see Table 1) [3], [4].

**Table 1:** Degrees of kidney damage severity.

Degree of damage	Type of damage	Description
І	Contusion	Contusion, micro or macro hematuria.
	Hematoma	Subcapsular, non-enlarging, without rupture of the parenchyma.
ІІ	Hematoma	Non-enlarging perirenal hematoma, limited by paranephric.
	Rupture	<1.0 cm in the depth of the kidney cortex, without extravasation.
ІІІ	Rupture	>1.0 cm in the depth of the kidney cortex, without rupture of the hollow system or extravasation of urine
ІV	Rupture	Rupture of the parenchyma, passing through the cortical layer, the medullary layer and the hollow system
	Vascular	Damage to the main artery or vein of the kidney with accompanying bleeding
V	Rupture	Complete crushing of the kidney
	Vascular	A detachment of the renal pedicle, completely devascularizing the kidney

In accordance with the recommendations of the World Society of Emergency Surgery (WSES) and the American Association for Traumatic Surgery (AAST) [2], the classification divides kidney damage into four classes, taking into account the hemodynamic status.

• Class I WSES - hemodynamically stable injured, AAST grade I–II, blunt and penetrating injuries.
• Class II WSES - hemodynamically stable, AAST grade III, blunt and penetrating injuries.
• Class III WSES - hemodynamically stable, blunt and penetrating injuries of the IV–V degree of AAST, parenchymal injuries of any degree with arterial dissection or occlusion.
• Class IV WSES - hemodynamically unstable AAST grades I-V, blunt and penetrating injuries.

**Fig. 1:**
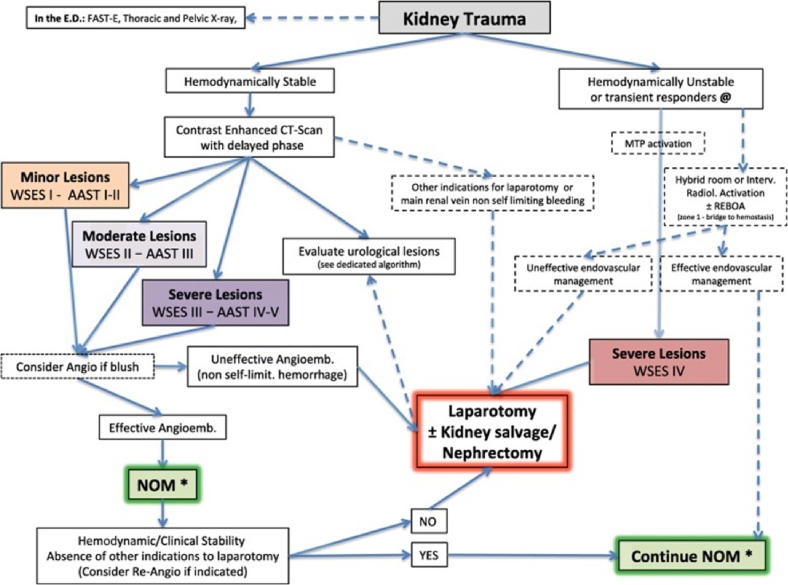
Surgical tactics for kidney damage.

Based on the current classification, the WSES and AAST used the renal injury management algorithm, shown in Fig. 1.

The choice of diagnostic method depended on the patient's hemodynamic status. FAST sonography is effective and fast for detecting intra-abdominal free fluid but has low sensitivity and specificity for kidney injury. Delayed urographic phase with contrast-enhanced computed tomography has been the gold standard in hemodynamically stable patients after blunt or penetrating trauma when there is suspicion of renal or urinary tract injury.

Diagnostic laparoscopy was of the greatest value. A visualized retroperitoneal hematoma in the projection of the kidneys was an indication for revision of the retroperitoneal space. Uncontrolled life-threatening bleeding with destruction of the renal parenchyma or detachment of the renal pedicle and a pulsatile enlarging of retroperitoneal hematoma or injury to the renal vein without self-limiting bleeding, was an indication for nephrectomy.

We performed two laparoscopic nephrectomies due to gunshot shrapnel damage to the kidney in a military mobile hospital at the II level of medical support. The entrance to the gun hole was tamponed beforehand. In one injured person, laparoscopy was performed in a horizontal position, in the other - in a lateroposition on the left side. The degree of kidney damage was assessed according to the AAST classification. The damaged kidney was removed through a separate incision above the bosom. Operative intervention ended with drainage of the retroperitoneal space.

## 3 Presentation of case

**Fig. 2:**
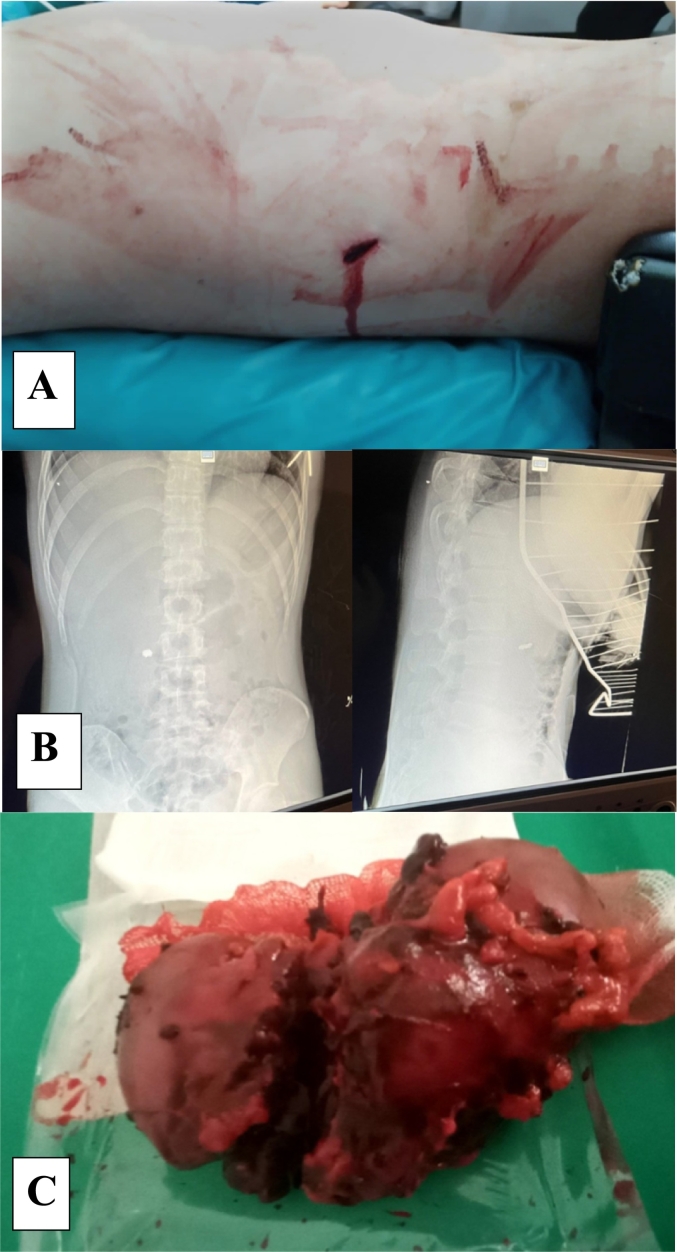
Wounded B., (A - The entrance to fire hole of the right lumbar area; B - X-ray in the direct and lateral projection of the organs of the abdominal cavity - presence of a foreign body - a metal fragment in the right side of the abdomen; C - macropreparation, right kidney removed after its damage by gunshot fragment - V degree according to the AAST classification.)

Wounded B., 21 years old was taken to a military mobile hospital. Complaints of pain in the injured areas of the right shoulder and right lumbar region. According to the anamnesis, he came under artillery fire (Fig. 2A).

Upon admission to the anti-shock ward, the condition was of moderate severity. Conscious, pale skin, complaints of pain in the areas of injuries, general weakness. Pulse – 112/min, blood pressure 100/60 mmHg, bandages in the area of the right shoulder and right lumbar area were soaked with blood. Anti-shock measures were carried out, a catheter was placed in the jugular vein, hemotransfusion. Laboratory examination – hemoglobin 76 g/l, erythrocytes – 2.8 × 10 g/l. FAST protocol is negative. A metal fragment was diagnosed radiologically in the abdominal cavity on the right (Fig. 2B).

After anti-shock measures, we decided to perform a diagnostic laparoscopy under endotracheal anesthesia. A retroperitoneal hematoma was detected in the projection of the right kidney. Opening and removal of the retroperitoneal hematoma on the right was performed laparoscopically, revealing a gunshot fragment injury of the right kidney of the V degree of severity according to AAST (Fig. 2C). We performed laparoscopic nephrectomy on the right, removing the metal fragment, and drainaged the retroperitoneal space. The operative time - 115 min. The volume of estimated blood loss – 1250 ml. External fixation apparatus was applied to the right shoulder. Primary surgical treatment of a gunshot through wound of the right shoulder, right lumbar region.

**Fig. 3:**
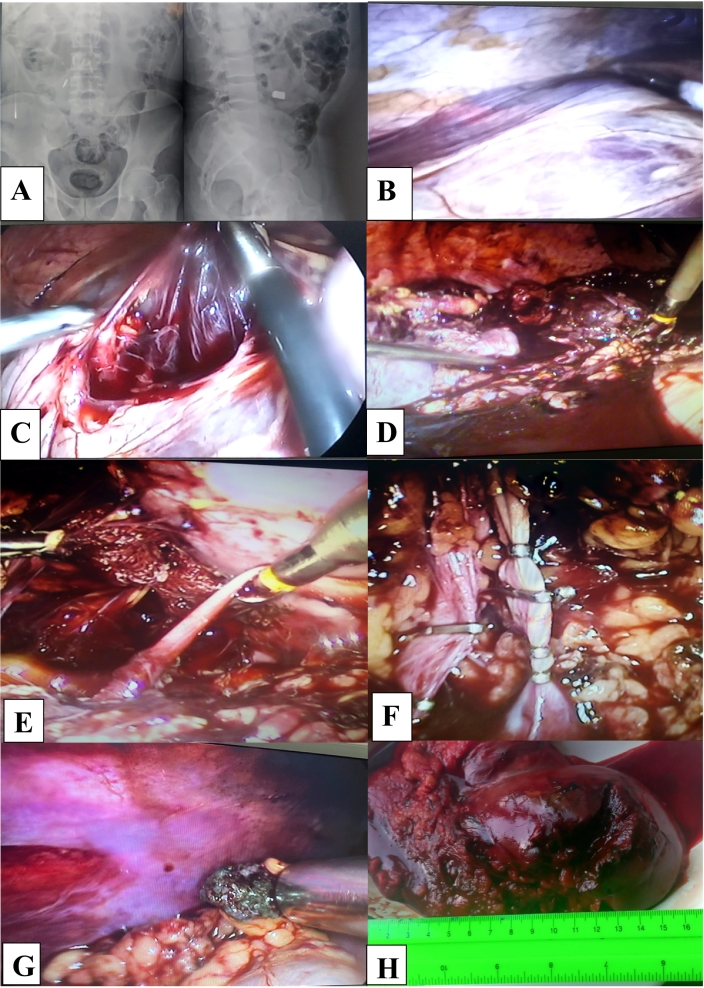
Wounded V., (A - X-ray in the direct and lateral projection of abdominal organs - presence of a foreign body - a metal fragment in the right side of the abdomen; B - Inspection laparoscopy – retroperitoneal hematoma on the right; C - Opening and removal of the retroperitoneal hematoma on the right; D - Bleeding from the right kidney with a firearm shrapnel damage; E - Visualization of the right ureter; F - Clipping of the renal artery and renal vein on the right; G - metal fragment was removed with a magnet instrument during laparoscopy; H - macropreparation, the right kidney was removed due to fire shrapnel damage - V degree according to the AAST classification).

Wounded V., 35 years old, was taken to the military mobile hospital by sanitary car transport 85 min after receiving the injury. Complaints of moderate pain in the right lumbar region at the site of injury, no other complaints. From the anamnesis, he came under mortar fire. A combat medic of the brigade provided the first aid. An aseptic bandage was applied to the gunshot wound of the right lumbar region. Nalbuphine solution - 2.0 ml. was administered intramuscularly. Upon admission to the anti-shock ward, he was conscious, complaining of pain in the area of the wound, general weakness. Pulse – 98/min, blood pressure 115/75 mmHg, the bandage in the area of the right lower back was moderately soaked with blood. The doctors carried out anti-shock measures, placed a jugular vein catheter, performing hemotransfusion. Laboratory examination – hemoglobin 84 g/l, erythrocytes – 3.0 × 10 g/l, Ht – 0.36. FAST protocol is negative. Radiologically, a metal fragment was diagnosed in the abdominal cavity on the right (Fig. 3A).

After anti-shock measures, a diagnostic laparoscopy was performed under endotracheal anesthesia (Fig. 3B, C).

We found grade V kidney injury according to AAST, performed laparoscopic nephrectomy on the right, removed a metal fragment of the right retroperitoneal space (Fig. 3D-H).

Drainage of the retroperitoneal space on the right. The operative time - 95 min. The volume of estimated blood loss – 850 ml. Primary surgical treatment of a gunshot wound in the right lumbar area.

**Fig. 4:**
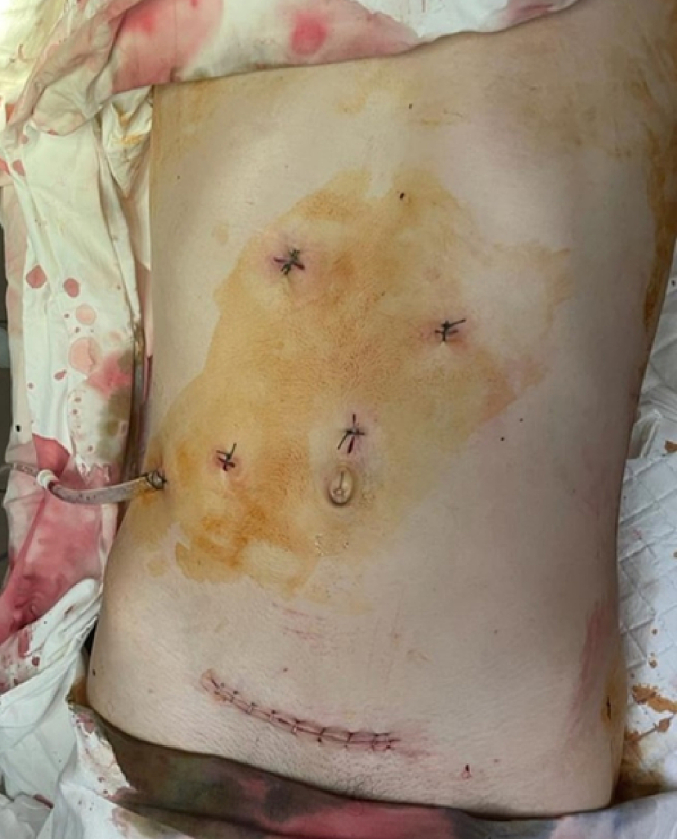
General view after laparoscopic nephrectomy on the right after a gunshot wound.

The wounded were extubated after the operations. On the first day, they were evacuated to another level of medical care. It should be noted that as on the first day, they were activated and could move around the ward independently. In one case, the drain was removed on the third day, in the other – on the 4 day. Right-sided lower lobe pneumonia was found in one wounded person in the postoperative period, which required additional conservative treatment and discharge on the sixteenth day. One wounded person was discharged from the department after removal of stitches on the eighth day. The wounded were sent for rehabilitation treatment. There were no complications in both wounded patients during the 30-day follow-up period after the operation. A better cosmetic effect was noted after laparoscopic operations (Fig. 4).

## 4 Discussion

Laparoscopic nephrectomy in gunshot damage to the kidney was characterized by presence of a retroperitoneal tense hematoma. When opened, there were signs of bleeding from the kidney parenchyma, difficulty of anatomical visualization of anatomical structures - ureter, renal artery and vein.

To determine the degree of damage to the kidney, we need its full mobilization. In case of massive bleeding, we should use dense tamponade of the wound site with napkins with constant aspiration. Electrocoagulation of the wound site at IV and V degrees according to the AAST classification does not stop the bleeding. Moreover, the renal artery and vein with their clipping must be rapidly visualized.

## 5 Conclusion

It is possible to perform endovideosurgical operations - laparoscopic nephrectomy in combat kidney injury at a military mobile hospital with available high-tech equipment at the II level of medical support (Role II), thus bringing highly specialized care closer to the wounded. A trained and experienced surgical team is needed to perform such operations. An important point is to determine the presence of a functionally healthy other kidney, visualize the anatomical structures of the damaged kidney and quickly clip the renal artery and vein, to remove foreign bodies – bullets and fragments. The possibility to perform laparoscopic organ-sparing operations in case of combat kidney injury, taking into account the indications and the degree of its damage according to the AAST classification, requires further scientific research.

## Ethical approval

This study has been exempted from ethical approval by our institution.

## Sources of funding

Not applicable.

## Consent

Written informed consent was obtained from the patient for publication of this case report and accompanying images. A copy of the written consent is available for review by the Editor-in-Chief of this journal on request.

## Author contribution

- Gumeniuk K., study concept and design, data collection, data analysis and interpretation, writing the paper, artwork editing, bibliographic research.

- Lurin I., data collection, data analysis and interpretation, writing the paper, bibliographic research.

- Savytskyi O., data collection, data analysis and interpretation, grammar correction.

- Nehoduiko V., data analysis and interpretation, writing the paper, artwork editing.

- Makarov V., data collection, data analysis and interpretation.

- Smolianyk K., data analysis and interpretation, writing the paper, artwork editing, visualization.

## Guarantor

Smolianyk Kostiantyn.

## Registration of research studies

Not applicable.

## Declaration of competing interest

Not applicable.
